# Phosphodiesterase inhibition restores hypoxia-induced cerebrovascular dysfunction subsequent to improved systemic redox homeostasis: A randomized, double-blind, placebo-controlled crossover study

**DOI:** 10.1177/0271678X251313747

**Published:** 2025-01-25

**Authors:** Benjamin S Stacey, Christopher J Marley, Hayato Tsukamoto, Tony G Dawkins, Thomas S Owens, Thomas A Calverley, Lewis Fall, Angelo Iannetelli, Ifan Lewis, James M Coulson, Mike Stembridge, Damian M Bailey

**Affiliations:** 1 Neurovascular Research Laboratory, Faculty of Life Sciences and Education, 6654 University of South Wales, Pontypridd, UK; 2 Faculty of Sport Sciences, Waseda University, Shinjuku, Tokyo, Japan; 3 School of Health and Exercise Sciences, University of British Columbia, Kelowna, Canada; 4 University Hospital Wales, Cardiff, UK; 5 Clinical Pharmacology, Therapeutics & Toxicology, Cardiff University, Cardiff, UK; 6 Cardiff School of Sport and Health Sciences, Cardiff Metropolitan University, Cardiff, UK

**Keywords:** Cerebral autoregulation, cerebral blood flow, cerebrovascular reactivity, nitric oxide, oxidative stress, phosphodiesterase inhibition

## Abstract

To what extent sildenafil, a selective inhibitor of the type-5 phosphodiesterase modulates systemic redox status and cerebrovascular function during acute exposure to hypoxia remains unknown. To address this, 12 healthy males (aged 24 ± 3 y) participated in a randomized, placebo-controlled crossover study involving exposure to both normoxia and acute (60 min) hypoxia (Fi ${\text{O}}_{2}$ = 0.14), followed by oral administration of 50 mg sildenafil and placebo (double-blinded). Venous blood was sampled for the ascorbate radical (A^•−^: electron paramagnetic resonance spectroscopy) and nitric oxide metabolites (NO: ozone-based chemiluminescence). Transcranial Doppler ultrasound was employed to determine middle cerebral artery velocity (MCAv), cerebral delivery of oxygen ${\text{(CDO}}_{2}\text{),}$ dynamic cerebral autoregulation (dCA) and cerebrovascular reactivity to hypo/hypercapnia (CVR_CO2HYPO/HYPER_). Cortical oxyhemoglobin (cO_2_Hb) and oxygenation index (OI) were assessed using pulsed continuous wave near infra-red spectroscopy. Hypoxia decreased total plasma NO (*P = *0.008), ${\text{CDO}}_{2}$ (*P* = <0.001) and cO_2_Hb (*P = *0.005). In hypoxia, sildenafil selectively reduced A^•−^ (*P = *0.018) and MCA_V_ (*P = *0.018), and increased dCA metrics of low-frequency phase (*P = *0.029) and CVR_CO2HYPER_ (*P = *0.007) compared to hypoxia-placebo. Collectively, these findings provide evidence for a PDE-5 inhibitory pathway that enhances select aspects of cerebrovascular function in hypoxia subsequent to a systemic improvement in redox homeostasis and independent of altered vascular NO bioavailability.

## Introduction

Sildenafil was primarily developed for the treatment of hypertension and angina pectoris^1^ owing to its highly selective ability to inhibit type-5 phosphodiesterase (PDE-5) and modulate the nitric oxide (NO)/cyclic guanosine monophosphate (cGMP) pathway.^2^ Sildenafil has since been of interest for providing neuroprotection,^3–5^ in light of its ability to cross the blood-brain barrier (BBB)^6^ and increase the expression of PDE-5 mRNA and protein in neurons, glial cells and cerebrovascular endothelial cells^7–9^ while also activating angiogenesis and neurogenesis.^10–13^ While limited data exists, sildenafil has been shown to increase basal cerebral blood flow (CBF) in healthy humans^14,15^ and more recently, lower the risk of Alzheimer’s Disease particularly in those most frequently issued prescriptions for erectile dysfunction.^16^

The vascular protective benefits of sildenafil are likely attributed to its direct and indirect impact on redox status. The selective inhibition of PDE-5 has been shown to enhance endogenous antioxidant defenses given observed increases in the expression of erythrocyte superoxide dismutase and catalase activity^17,18^ and inhibition of nicotinamide adenine dinucleotide phosphate (NADPH) activity,^2,19^ collectively effecting a reduction in free radical and associated reactive oxygen species (ROS) formation preceding liberation of vascular NO.^17^ Sildenafil acts to increase the biological effect of NO by attenuating cGMP hydrolysis,^20^ subsequently increasing intracellular cGMP concentration that mediates the NO-induced activation of cGMP-dependent protein kinases and ion channels.^21^

Exposure to the hypoxia of high-altitude promotes local elevations in oxidative-nitrosative stress (OXNOS), reflected by a free radical-mediated reduction in vascular NO bioavailability.^22^ This has been further associated with cerebrovascular dysfunction, namely attenuated vasoreactivity to hypercapnia^23^ and select metrics of dynamic cerebral autoregulation (dCA).^23,24^ While sildenafil has been used prophylactically to attenuate the hypoxia-induced elevation in pulmonary artery systolic pressure, the primary risk factor for high-altitude pulmonary oedema,^25,26^ there is only one study that has explored its impact on the cerebrovasculature during inspiratory hypoxia. Accordingly, Chan, et al.^18^ reported a single dose (50 mg) of sildenafil increased cerebral oxygenation on days 1 and 3 following rapid ascent to 3,480 m. However, to what extent sildenafil could recover the aforementioned hypoxia-induced cerebrovascular dysfunction^23,24^ remains to be established.

In light of these findings, we conducted a randomized, double-blind, placebo-controlled crossover trial utilizing a functionally integrative translational approach to examine the impact of PDE-5 inhibition on hypoxia-induced systemic OXNOS and corresponding implications for cerebrovascular function. We hypothesized that sildenafil would attenuate hypoxia-induced systemic OXNOS and promote improvements in select indices of cerebral hemodynamic function.

## Methods

### Ethics

The experimental protocol was approved by the Research Ethics Committees of the University of South Wales (#17070LSE) and Cardiff Metropolitan University (#16/8/01 R). All experimental procedures were carried out in accordance with the most recent (7^th^) amendment of the Declaration of Helsinki of the World Medical Association,^27^ with the exception that it was not registered in a publicly accessible database, with verbal and written informed consent obtained from all participants.

### Participants

Twelve healthy, physically active young (26 ± 12 y) males with a body mass index (BMI) of 26 ± 3 kg·m^−2^ were recruited into the study (see power calculations in statistical analysis). All participants were free of medication, non-smokers and abstained from taking nutritional supplements, including oral antioxidants and anti-inflammatories. Participants were specifically asked to refrain from physical activity, caffeine and alcohol for a period of 48 h prior to formal experimentation, to minimize biological variation.^24^ They were also encouraged to follow a low-nitrate/nitrite ( ${\text{NO}}_{3}^{-}/{\text{NO}}_{2}^{-}$ ) diet for 96 h prior to the study, with specific instructions to avoid fruits, salads and cured meats.^28^

### Design

**Figure 1.**
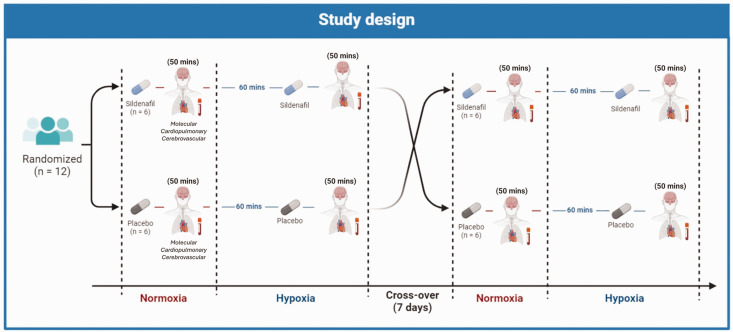
Study design. The study adopted a randomized, double-blind, placebo-controlled crossover design. All participants completed two trials (sildenafil and placebo) and were exposed to both normoxia $({\text{FiO}}_{2}$ = 0.21) and normobaric hypoxia $({\text{FiO}}_{2}$ = 0.14) during both visits. Participants were administered either 50 mg (oral) sildenafil or 50 mg placebo which was followed by a 50 min resting period in normoxia before data collection (blood sample, cardiopulmonary and cerebrovascular assessments). Participants were then exposed to hypoxia for 60 min. A second tablet of either 50 mg sildenafil or 50 mg placebo was then administered followed by another 50 min rest period prior to a second bout of data collection. Each trial was separated by a 7-day recovery period.

The study adopted a randomized, double-blind, placebo-controlled crossover design (Figure 1). All participants completed two trials (oral administration of sildenafil and placebo) and were exposed to both normoxia $({\text{FiO}}_{2}$ = 0.21) and normobaric hypoxia $({\text{FiO}}_{2}$ = 0.14) during both visits. Measurements were conducted in a ∼120 m^3^ environmental chamber maintained at 21°C and 50% relative humidity (Weiss Technik UK Limited, Ebbw Vale, UK). Participants attended the laboratory following a 12 h overnight fast and ingested a tablet containing either 50 mg sildenafil (sildenafil citrate, [Viagra], Pfizer, UK) or 50 mg placebo (Placebo-lactate, Placebo-world, UK) which had an identical appearance, taste and smell in a double-blind, randomized manner by the toss of a fair coin. Following a 50 min normoxic resting period (allowing for sildenafil to reach peak plasma concentration^29^) venous blood samples were obtained, followed immediately by cardiopulmonary and cerebrovascular measurements. Participants then re-entered the environmental chamber (hypoxia) and rested (seated) for 60 min. A second tablet of either 50 mg sildenafil or 50 mg placebo was subsequently administered (to maintain peak plasma sildenafil concentration) followed by another 50 min rest prior to a second bout of blood/hemodynamic data collection. Each trial was separated by a 7-day washout period.

## Measurements

### Molecular function

Blood was collected from an indwelling cannula into Vacutainers® (Becton, Dickinson and Company, Oxford, UK) before centrifugation at 600 *g* (4°C) for 10 min. Plasma (K-EDTA) and red blood cell (RBC) supernatant were decanted into cryogenic vials (Nalgene® Labware, Thermo Fisher Scientific Inc, Waltham, MA, USA), immediately snap-frozen in liquid nitrogen and subsequently stored at −80°C. Prior to batch analysis, each sample were thawed at 37°C in the dark for 3 min. Whole blood was also assayed for hemoglobin (Hb) and hematocrit (Hct). Hb was measured photometrically (HemoCue 201+, Radiometer, UK) and Hct was prepared via ultracentrifugation (Hawksley and Sons Ltd, Sussex, UK) and measured using a Hawksley Micro Hematocrit Reader (Hawksley and Sons Ltd, Sussex, UK).

#### Free radicals

The ascorbate free radical (A^•−^) was employed as a direct measure of global free radical formation.^30,31^ Plasma (1 mL) was injected directly into a high-sensitivity multiple-bore sample cell (AquaX, Bruker Daltonics Inc., Billerica, MA, USA) housed within a TM_110_ cavity of an EPR spectrometer operating at X-band (9.87 GHz). Samples were recorded by cumulative signal averaging of 10 scans using the following instrument parameters: resolution, 1024 points: microwave power, 20 mW; modulation amplitude, 0.65 G; receiver gain, 2 × 10^5^; time constant, 40.96 ms; sweep rate, 0.14 G/s; sweep width, 6 G; centre field, 3486 G. Spectra were filtered identically (moving average, 15 conversion points) using WINEPR software (Version 2.11, Bruker, Karlsruhe, Germany) and the double integral of each doublet was determined using commercial software (OriginLab Corps, MA, USA). The intra- and inter-assay CVs were both *<*5%.^31^

#### NO metabolites

Ozone-based chemiluminescence (Sievers NOA 280i, Analytix Ltd, Durham, UK) was employed to detect NO liberated from plasma and RBC samples via chemical reagent cleavage as previously described in detail.^32,33^ This facilitated detection of total plasma (combined concentration of nitrite [ ${\text{NO}}_{2}^{-}]$ + *S*-nitrosothiols [RSNO]) and total RBC-bound (combined concentration of ${\text{NO}}_{2}^{-}$ + *S*-nitrosohemoglobin [SNO-Hb] and iron nitrosylhemoglobin [HbNO]) NO metabolites. Plasma (200 µL) was injected into tri-iodide (I_3_) reagent for the measurement of total plasma NO. The original I_3_ reagent was subsequently modified with the addition of potassium hexacyanoferrate [K_3_Fe^III^(CN)_6_] to limit Hb/cell free heme [Fe(II)] auto-capture of NO for the analysis of total RBC-bound NO.^34^ Briefly, RBC samples were lysed 1:4 with EDTA (0.5 mM final concentration, pH 7.0), incubated for 5 min on ice and subsequently injected (400 uL) into the modified I_3_ reagent. The signal output (in mV) from the analyzer was plotted against time using Origin 8 (OriginLab Corps, Massachusetts, USA) and smoothed using a 150-point averaging algorithm. The Peak Analysis package was used to calculate area under the curve (mV/s), that was subsequently converted to a concentration, using standard curves of known concentrations of sodium nitrite, measured at identical injection volumes. The intra-assay and inter-assay coefficients of variation for plasma and RBC-bound NO metabolites were <10%.^32^

### Cardiopulmonary function

Beat-by-beat arterial blood pressure was assessed via finger photoplethysmography and arterial volume clamping (Finometer PRO, Finapres Medical Systems, Amsterdam, Netherlands) and used to calculate mean arterial blood pressure (MAP) after calibrating values to the average of three automated brachial blood pressure measurements (Life Source, A&D Medical, model: UA767FAM), taken over a 5 min resting baseline period. Heart rate (HR) was assessed using a 3-lead electrocardiogram (ECG, ADI BioAmp ML132, ADInstruments, Colorado Springs, CO, USA). Respiratory flow was measured with a pneumotachometer (model HR 800 L, Hans Rudolph, Shawnee, KS) and expired gases (end-tidal partial pressures of ${\text{CO}}_{2}$ and ${\text{O}}_{2}$ [P_ET_C ${\text{O}}_{2}$ and P_ET_ ${\text{O}}_{2}$ ]) were sampled continuously via capnography (model ML206, ADInstruments, Colorado Springs, CO, USA). Stroke volume (SV) was estimated from the arterial blood pressure waveform using the modelflow (MF) method.^35^ Cardiac output ( $\dot{Q}$ ) was calculated mathematically as the product of HR and SV. Total peripheral resistance (TPR) was calculated as $\frac{\text{MAP}\;\text{(mmHg)}}{\dot{Q}\;\mathrm{(L∙min}\text{-}1\text{)}}$ . Peripheral oxygen saturation ( ${\text{SpO}}_{2})\;$ was measured using fingertip pulse oximetry (Nonin 9550 Onyx II, Nonin Medical, Inc., Plymouth, MI, USA). All cardiopulmonary variables were sampled continuously at 1 kHz using an analogue-to-digital converter (Powerlab, 16/30; ADInstruments, Colorado Springs, CO, USA) and data were interfaced with LabChart (Version 7.3), and analyzed offline.

### Cerebrovascular function

#### Cerebral haemodynamics

The proximal segment of the right middle cerebral artery (MCA) was insonated using a 2 MHz-pulsed transcranial Doppler (TCD) ultrasound system (Multi-Dop X4; DWL Electroniche Systeme, Singen, Germany). Following standardized search techniques,^36^ the Doppler probe was secured over the middle trans-temporal window using a custom fit headband device (Spencer Technologies) to securely insonate the MCA and measure blood velocity (MCAv). Indices of cerebrovascular conductance (CVCi) and cerebrovascular resistance (CVRi) were calculated as mean MCAv/MAP and MAP/mean MCAv, respectively. Pulsatility index (PI) was calculated as: systolic MCAv−diastolic MCAv/mean MCAv and subsequently normalized to the prevailing MAP (PI_Norm_ = PI/MAP). Cerebral ${\text{O}}_{2}$ delivery (CD ${\text{O}}_{2}$ ) was calculated as mean MCAv × arterial oxygen content (CaO_2_) where CaO_2_ was estimated as: caO_2_ (mL/dL) = Hb (g/dL)×

1.34 $\;\left(\frac{\text{SaO}2\;(\%)}{100}\right).$ ^24^

#### Cerebral oxygenation

Pulsed continuous-wave near-infrared spectroscopy (NIRS, Oxymon Mk III; Artinis Medical Systems BV, Zetten, The Netherlands) was used to monitor changes in cerebral oxygenation at optical densities of 780 and 850 nm.^37^ One set of NIRS optodes (5.0 cm source detector spacing, consistent with recent recommendations) was placed on the skin over the left frontal prefrontal cortical region of the forehead between Fp1 and F3, consistent with the anatomical landmarks of the International 10–20 system for EEG placement.^38^ Optodes were secured in place using double-sided adhesive tape and positioned underneath the custom fit headband device. Concentration changes (Δ) in oxyhemoglobin (O_2_Hb) and deoxyhemoglobin (HHb) were calculated using the modified Beer–Lambert Law^39^ incorporating a differential pathlength factor (DPF) of 5.93% for cerebral tissue.^40^ We also calculated the difference between Δ[O_2_Hb] and Δ[HHb] as a surrogate ‘oxygenation index’ (OI), consistent with NIRS studies that have also employed similar continuous-wave devices (Grassi *et al.* 2003). Finally, Δ[THb] was calculated as the sum of Δ[O2Hb] and Δ[HHb] signals and used as a surrogate for changes in regional blood volume (Van Beekvelt *et al.* 2001). All NIRS signals were normalized to reflect changes relative to a 5 min resting baseline control in normoxia following administration of the placebo (arbitrarily defined as 0 µmol·L^−1^). Signals were recorded at 50 Hz, displayed in real time and stored on a computer for offline analysis. A 60 s average at the end of a five min resting baseline was calculated in each condition.

#### Dynamic cerebral autoregulation (dCA)

Following 10 min of seated rest, 5-min segments of MAP and MCAv data were obtained for spectral analysis of spontaneous oscillations to assess dCA via transfer function analysis (TFA).^41^ Beat‐to‐beat MAP and MCAv mean signals were calculated across each cardiac cycle, linearly interpolated, and resampled at 4 Hz in accordance with formal recommendations of the Cerebrovascular Research Network.^42,43^ Spontaneous MAP and MCAv mean power spectrum density and the mean value of TFA coherence, gain, and phase were band averaged across the very low frequency (VLF: 0.02–0.07 Hz, 50 to 14.3-s cycles) and low frequency (LF: 0.07–0.2 Hz, 14.3 to 5-s cycles) ranges where CA is considered to be most operant.^41^ The squared coherence function reflects the fraction of output power (i.e., MCAv mean) that can be linearly related to the input power (i.e., MAP) at each frequency. Similar to a correlation coefficient, this value varies between 0 and 1 with 1 indicating that all the variability in MCA_V_ is linearly explained by the variability in MAP. To ensure that robust phase and gain estimates were entered for subsequent analysis, we averaged only those gain and phase (positive to eliminate wrap-around) values where the corresponding coherence was ≥0.34, applied to single harmonics only. An increase in gain and reduction in phase were taken to reflect reduced dCA, indicative of a more pressure-passive relationship between MAP and MCAv.^24^

#### Cerebrovascular reactivity to carbon dioxide (CVR_CO2_)

Following the 5 min resting baseline for TFA, the inspired air was rapidly changed to 5% C ${\text{O}}_{2}$ with 21% ${\text{O}}_{2}$ (balanced nitrogen) from a 200 L Douglas Bag attached to Falconia tubing (Cranleigh, UK) connected to the inspiratory port of a 2-way nonrebreathing valve (Hans Rudolph, 2400 series) for 3 min. Following 5 min of recovery breathing ambient air, participants were instructed to hyperventilate at 15 breaths/min for 3 minutes. From this, CVR_CO2_ to hypercapnia (CVR_CO2Hyper_)/hypocapnia (CVR_CO2Hypo_) were calculated as the percent change in MCAv from baseline per mmHg change in P_ET_C ${\text{O}}_{2}$ recorded during the final 30 s (average taken) of the respective challenge when steady-state had been achieved. We also derived the CVR_CO2Range_ as a useful indication of the cerebral circulation’s combined ability to respond to differential changes in C ${\text{O}}_{2}$ , calculated as the change in MCAv divided by the change in P_ET_C ${\text{O}}_{2}$ across the hypocapnia-hypercapnia range.

### Statistical analysis

#### Power calculations

Prospective power calculations were determined using G*Power (V.3.1.9.4). Assuming comparable differences (15%) and corresponding effect size (0.60) previously observed in basal plasma A^•−^ (η^2^ = 0.54)^44^ between normoxia and hypoxia, our primary end-outcome variable for OXNOS, the present study required a (minimum) sample size of 20 participants (10 per group) in order to achieve adequate power (1-β = 0.80 at P < 0.05). We chose to further inflate this by 20% during recruitment (n = 24 or 12 per group) given the potential for loss to follow-up or incomplete data collection.

#### Inferential analyses

Data were analyzed using the Statistics Package for Social Scientists (IBM SPSS Statistics Version 29.0). Shapiro-Wilk *W* tests (all P > 0.05) confirmed that all data sets were normally distributed. A 2-way (Inspirate: normoxia vs. hypoxia × Drug: placebo vs. sildenafil) repeated measures analysis of variance (ANOVA) was used to detect differences between conditions and presented alongside effect sizes (partial eta squared: η_p_^2^) and estimates of statistical power (β). Where interaction effects were detected, post-hoc comparisons were made using paired samples *t-*tests with Bonferroni correction. Significance was established at *P < *0.05 for all two-tailed tests and data expressed as mean ± standard deviation (SD).

## Results

### Compliance and randomization

A total of 12 participants completed both placebo and sildenafil trials with no loss to follow-up. Randomization led to 6 participants completing the sildenafil trial first with 6 participants completing the placebo trial first (Figure 1).

### Molecular function

**Figure 2.**
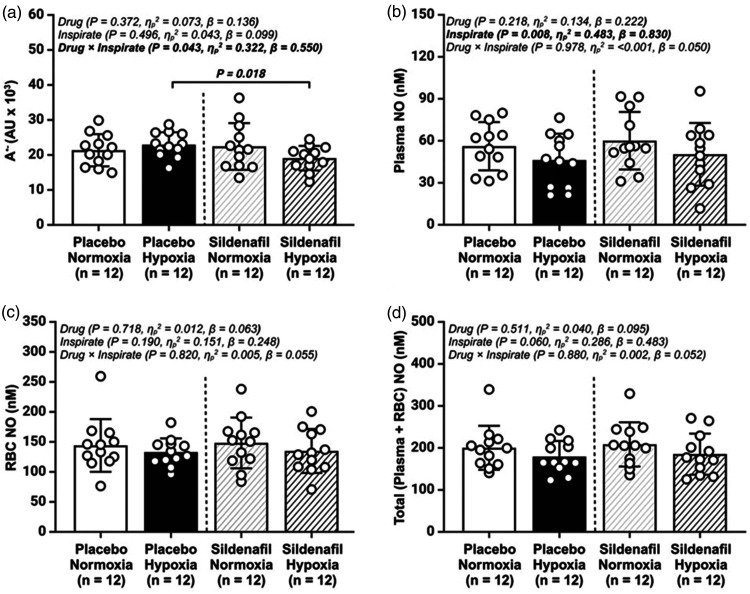
Basal metrics of systemic oxidative-nitrosative stress (OXNOS). (a) plasma ascorbate free radical (A^•−^); (b) total plasma nitric oxide (NO) concentration (nitrite + *S*-Nitrosothiols); (c) total red blood cell (RBC) NO (nitrite + *S*-Nitrosohemoglobin +hemoglobin-bound NO); and (d) total (plasma + total RBC) NO concentrations; AU, arbitrary units; Values are mean ± SD. n = 12 for all parameters.

During the placebo trial, hypoxia did not impact ${\text{A}}^{\text{·}-}$ . However, ${\text{A}}^{\text{·}-}$ was decreased following sildenafil in hypoxia, when compared to hypoxia-placebo (*P = *0.018, Figure 2). Hypoxia decreased plasma NO in both the placebo and sildenafil trials (*P = *0.008, Figure 2).

### Cardiopulmonary function

Hypoxia increased Hb (*P* = <0.001), Hct (*P = *0.004), $\dot{Q}$ (*P = *0.003) and $\dot{V}$ _E_ (*P* = <0.001), and reduced P_ET_ ${\text{O}}_{2}$ (*P* = <0.001), P_ET_C ${\text{O}}_{2}$ (*P* = <0.001), Sp ${\text{O}}_{2}$ (*P* = <0.001) and ca ${\text{O}}_{2}$ (*P* = <0.001) across the placebo and sildenafil trials. Sildenafil increased HR in the normoxia and hypoxia trial (*P = *0.001) but had no effect (*P > *0.05) on other cardiopulmonary variables (MAP, SV, $\dot{Q}$ , TPR, P_ET_ ${\text{O}}_{2}$ /C ${\text{O}}_{2}$ or $\dot{V}$ _E_) (Table 1).

**Table 1.** Cardiopulmonary function.

Drug	Placebo		Sildenafil	
Inspirate	Normoxia	Hypoxia	Normoxia	Hypoxia
*Cardiovascular*				
Hb (g · dL^−1^)	14.5 ± 1.5	14.8 ± 1.4	14.4 ± 1.2	14.7 ± 1.4
*Drug (P = 0.659, η_p_* ^2^ * = 0.018, β = 0.070);* ***Inspirate (P = <0.001, η_p_*** ^2^ *** = 0.749, β = 0.999)*** *; Drug × Inspirate (P = 0.761, η_p_* ^2^ * = 0.009, β = 0.059)*				
Hct (%)	45 ± 3	47 ± 2	46 ± 4	46 ± 4
*Drug (P = 0.779, η_p_* ^2^ * = 0.007, β = 0.058);* ***Inspirate (P = 0.005, η_p_*** ^2^ *** = 0.533, β = 0.897)*** *; Drug × Inspirate (P = 0.117, η_p_* ^2^ * = 0.208, β = 0.343)*				
SBP (mmHg)	133 ± 18	132 ± 11	135 ± 22	132 ± 27
*Drug (P = 0.823, η_p_* ^2^ * = 0.005, β = 0.055); Inspirate (P = 0.766, η_p_* ^2^ * = 0.008, β = 0.059); Drug × Inspirate (P = 0.837, η_p_* ^2^ * = 0.044, β = 0.054)*				
DBP (mmHg)	65 ± 10	64 ± 8	60 ± 11	63 ± 18
*Drug (P = 0.505, η_p_* ^2^ * = 0.041, β = 0.097); Inspirate (P = 0.828, η_p_* ^2^ * = 0.044, β = 0.055); Drug × Inspirate (P = 0.457, η_p_* ^2^ * = 0.051, β = 0.109)*				
MAP (mmHg)	88 ± 11	86 ± 6	85 ± 14	86 ± 20
*Drug (P = 0.721, η_p_* ^2^ * = 0.012, β = 0.063); Inspirate (P = 0.952, η_p_* ^2^ * = <0.001, β = 0.050); Drug × Inspirate (P = 0.720, η_p_* ^2^ * = 0.120, β = 0.063)*				
HR (bpm)	61 ± 10	65 ± 11	64 ± 9	72 ± 12
*Drug (P = 0.169, η_p_* ^2^ * = 0.164, β = 0.269);* ***Inspirate (P = 0.001, η_p_*** ^2^ *** = 0.653, β = 0.985)*** *; Drug × Inspirate (P = 0.278, η_p_* ^2^ * = 0.106, β = 0.181)*				
SV (mL)	102 ± 7	101 ± 3	102 ± 6	100 ± 1
*Drug (P = 0.708, η_p_* ^2^ * = 0.013, β = 0.064); Inspirate (P = 0.299, η_p_* ^2^ * = 0.097, β = 0.169); Drug × Inspirate (P = 0.866, η_p_* ^2^ * = 0.003, β = 0.053)*				
$\dot{Q}$ (L · min^−1^)	6.19 ± 0.96	6.61 ± 1.10	6.52 ± 0.96	7.18 ± 1.20
*Drug (P = 0.163, η_p_* ^2^ * = 0.169, β = 0.277);* ***Inspirate (P = 0.003, η_p_*** ^2^ *** = 0.564, β = 0.929);*** *Drug × Inspirate (P = 0.253, η_p_* ^2^ * = 0.117, β = 0.197)*				
TPR (mmHg · min · L^−1^)	14.70 ± 3.91	13.55 ± 3.29	13.39 ± 3.60	12.25 ± 3.43
*Drug (P = 0.198, η_p_* ^2^ * = 0.146, β = 0.240); Inspirate (P = 0.077, η_p_* ^2^ * = 0.256, β = 0.428); Drug × Inspirate (P = 0.996, η_p_* ^2^ * = <0.001, β = 0.050)*				
*Pulmonary*				
SpO_2_ (%)	99 ± 1	88 ± 4	99 ± 1	90 ± 2
*Drug (P = 0.210, η_p_* ^2^ * = 0.139, β = 0.229);* ***Inspirate (P = <0.001, η_p_*** ^2^ *** = 0.919, β = 1.000*** *); Drug × Inspirate (P = 0.199, η_p_* ^2^ * = 0.145, β = 0.239)*				
caO_2_ (mL · dL^−1^)	19.8 ± 2.0	18.2 ± 2.4	19.6 ± 1.5	18.4 ± 1.8
*Drug (P = 0.990, η_p_* ^2^ * = <0.001, β = 0.050);* ***Inspirate (P = <0.001, η_p_*** ^2^ *** = 0.901, β = 1.000)*** *; Drug × Inspirate (P = 0.289, η_p_* ^2^ * = 0.102, β = 0.175)*				
$\dot{V}$ _E_ (L · min^−1^)	19 ± 6	26 ± 4	18 ± 5	26 ± 4
*Drug (P = 0.989, η_p_* ^2^ * = <0.001, β = 0.050);* ***Inspirate (P = <0.001, η_p_*** ^2^ *** = 0.915, β = 1.000)*** *; Drug × Inspirate (P = 0.104, η_p_* ^2^ * = 0.380, β = 0.365)*				
P_ET_O_2_ (mmHg)	89 ± 4	51 ± 3	90 ± 5	50 ± 4
*Drug (P = 0.729, η_p_* ^2^ * = 0.011, β = 0.062);* ***Inspirate (P = <0.001, η_p_*** ^2^ *** = 0.995, β = 1.000)*** *; Drug × Inspirate (P = 0.121, η_p_* ^2^ * = 0.205, β = 0.336)*				
P_ET_CO_2_ (mmHg)	41 ± 3	38 ± 3	41 ± 3	38 ± 3
*Drug (P = 0.588, η_p_* ^2^ * = 0.126, β = 0.062);* ***Inspirate (P = <0.001, η_p_*** ^2^ *** = 0.995, β = 1.000)*** *; Drug × Inspirate (P = 0.825, η_p_* ^2^ * = 0.205, β = 0.336)*				

Values are mean ± SD; SBP: systolic blood pressure; DBP: diastolic blood pressure; MAP: mean arterial pressure; HR: heart rate; SV: stroke volume: $\dot{Q}$ : cardiac output; TPR: total peripheral resistance; Hb: hemoglobin; Hct: hematocrit; Sp ${\text{O}}_{2}$ : arterial oxyhemoglobin saturation; *ca* ${\text{O}}_{2}$ : arterial oxygen content; $\dot{V}$ _E_: minute ventilation; P_ET_ ${\text{O}}_{2}$ /P_ET_C ${\text{O}}_{2}$ : end-tidal partial pressure of oxygen/carbon dioxide. n = 12 for all parameters.

### Cerebrovascular function

**Figure 3.**
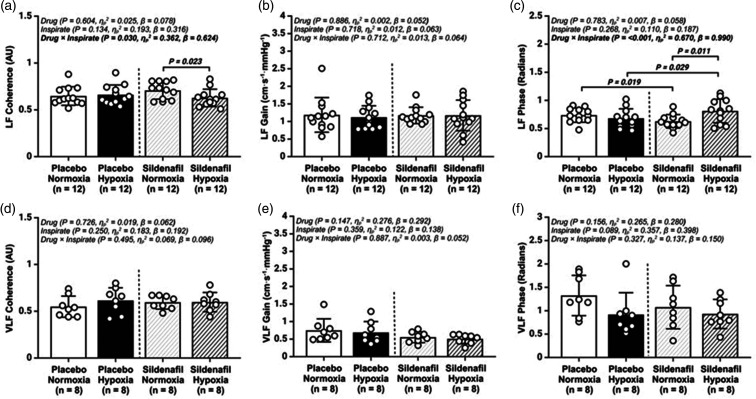
Transfer function analysis of the cerebral pressure–flow relationship during spontaneous oscillations in mean arterial blood pressure (MAP) and middle cerebral artery velocity (MCAv). (a) low frequency (LF) Coherence; (b) LF Gain; (c) LF Phase; (d) very low frequency (VLF) Coherence; (e) VLF Gain and (f) VLF Phase; AU, arbitrary units; Values are mean ± SD. n = 12 for LF parameters, whereas n = 8 for VLF data.

**Figure 4.**
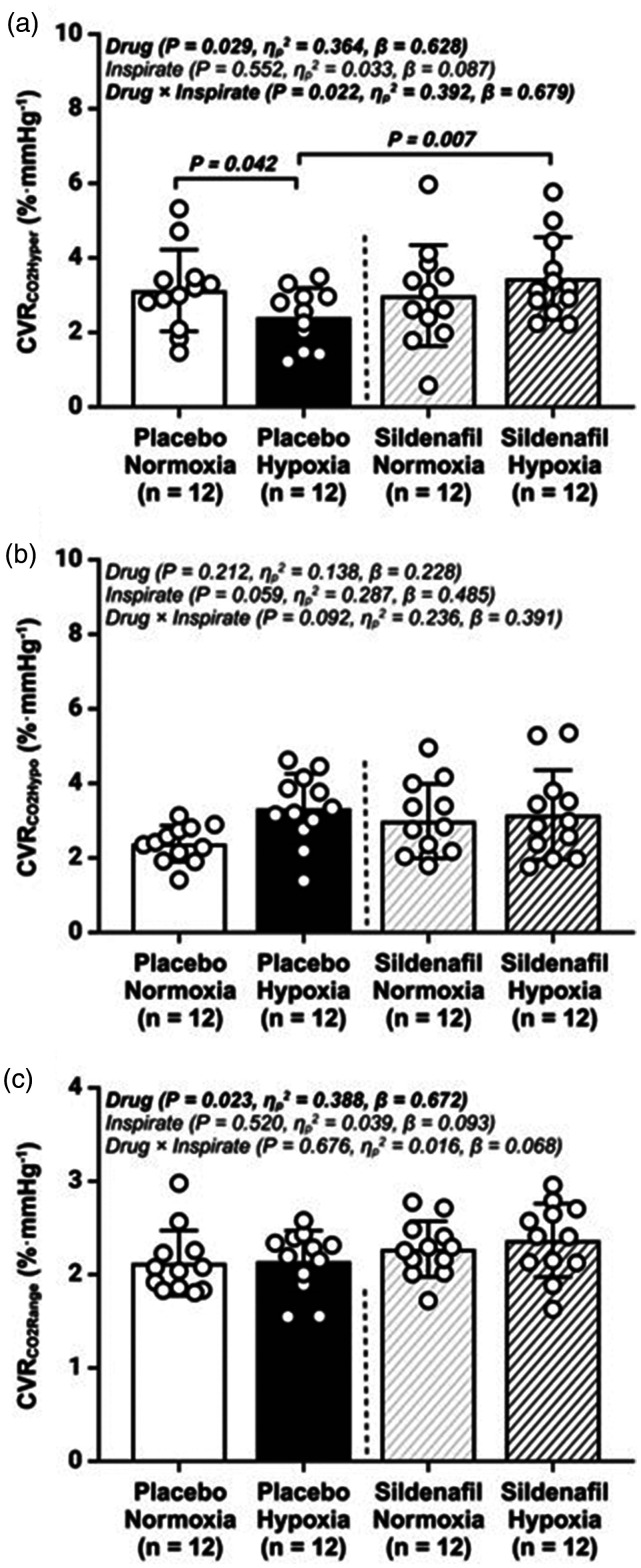
Cerebrovascular reactivity to carbon dioxide (CVR_CO2_). (a) cerebrovascular reactivity to hypercapnia (CVR_CO2Hyper_); (b) cerebrovascular reactivity to hypocapnia (CVR_CO2Hypo_) and (c) cerebrovascular reactivity to carbon dioxide range (CVR_CO2Range_); Values are mean ± SD. n = 12 for all parameters.

***Haemodynamics:*** Hypoxia reduced CD ${\text{O}}_{2}$ across both placebo and sildenafil trials (*P = *<0.001, Table 2). Compared to hypoxia-placebo and normoxia-sildenafil, hypoxia-sildenafil decreased MCA_V_ (*P = *0.018 and *P = *0.011, respectively; Table 2). ***Cortical oxygenation:*** As anticipated, hypoxia reduced cO_2_Hb (*P = *0.005) and OI (*P* = <0.001), whereas cHHb increased (P = 0.025) across both placebo and sildenafil trials (Table 2). ***Autoregulation:*** A total of 4 participants were excluded from the VLF estimates for gain and phase, owing to a VLF coherence <0.34. LF phase was increased under hypoxia-sildenafil when compared to the placebo-hypoxia (*P = *0.029; Figure 3(c)) and normoxia-sildenafil (*P = *0.011; Figure 3(c)) trials. In normoxia, sildenafil reduced LF phase (*P = *0.019; Figure 3(c)) when compared to the placebo trial. LF coherence was also reduced in the hypoxia-sildenafil (P = 0.023; Figure 3(a)). Despite differences in mean MCA_V_ across conditions, no differences were observed for normalized gain in either the VLF or LF range. ***Vasoreactivity:*** CVR_CO2HYPER_ was lower in the hypoxia-placebo trial when compared to the normoxia-placebo trial (2.2 ± 1.1 vs. 3.1 ± 1.1%·mmHg^−1^; *P = *0.042; Figure 4(a)). However, hypoxia-sildenafil increased CVR_CO2HYPER_ when compared to the hypoxia-placebo trial (3.4 ± 1.1 vs. 2.2 ± 1.1%·mmHg^−1^; *P = *0.007; Figure 4(a)). CVR_CO2RANGE_ was higher in the sildenafil trials in both normoxia and hypoxia (P = 0.023), when compared to the placebo trials (Figure 4(c)). No differences were observed for CVR_CO2HYPO_ or CVR_CO2RANGE_ (all p > 0.05; Figure 4(b) and (c)).

**Table 2.** Cerebrovascular function.

Drug	Placebo		Sildenafil	
Inspirate	Normoxia	Hypoxia	Normoxia	Hypoxia
*Cardiovascular*				
sMCA_V_ (cm·s^−1^)	114 ± 18	115 ± 18	121 ± 21	109 ± 16†
*Drug (P = 0.748, η_p_* ^2^ * = 0.010, β = 0.061* ***); Inspirate (P = 0.020, η_p_*** ^2^ *** = 0.400, β = 0.695); Drug × Inspirate (P = 0.024, η_p_*** ^2^ *** = 0.385, β = 0.666)***				
dMCA_V_ (cm·s^−1^)	36 ± 7	35 ± 6	34 ± 11	30 ± 7
*Drug (P = 0.164, η_p_* ^2^ * = 0.168, β = 0.275); Inspirate (P = 0.069, η_p_* ^2^ * = 0.270, β = 0.452); Drug × Inspirate (P = 0.162, η_p_* ^2^ * = 0.169, β = 0.278)*				
MCA_V_ (cm·s^−1^)	62 ± 10	62 ± 10	63 ± 12	57 ± 8*†
*Drug (P = 0.318, η_p_* ^2^ * = 0.091, β = 0.160);* ***Inspirate (P = 0.018, η_p_*** ^2^ *** = 0.413, β = 0.717); Drug × Inspirate (P = 0.036, η_p_*** ^2^ *** = 0.342, β = 0.587)***				
PI (AU)	1.26 ± 0.14	1.29 ± 0.12	1.38 ± 0.30	1.39 ± 0.22
*Drug (P = 0.163, η_p_* ^2^ * = 0.169, β = 0.277); Inspirate (P = 0.471, η_p_* ^2^ * = 0.048, β = 0.105); Drug × Inspirate (P = 0.600, η_p_* ^2^ * = 0.026, β = 0.078)*				
CVRi (mmHg·cm·s^−1^)	1.43 ± 0.20	1.43 ± 0.24	1.38 ± 0.30	1.55 ± 0.45
*Drug (P = 0.670, η_p_* ^2^ * = 0.017, β = 0.069); Inspirate (P = 0.345, η_p_* ^2^ * = 0.081, β = 0.147); Drug × Inspirate (P = 0.267, η_p_* ^2^ * = 0.111, β = 0.188)*				
CVCi (cm·s^−1^·mmHg^−1^)	0.71 ± 0.10	0.72 ± 0.12	0.76 ± 0.21	0.70 ± 0.22
*Drug (P = 0.666, η_p_* ^2^ * = 0.018, β = 0.069); Inspirate (P = 0.544, η_p_* ^2^ * = 0.034, β = 0.088); Drug × Inspirate (P = 0.402, η_p_* ^2^ * = 0.064, β = 0.125)*				
${\text{CDO}}_{2}$ (mL·cm·s^−1^)	1233 ± 240	1119 ± 220	1239 ± 240	1042 ± 168
*Drug (P = 0.379, η_p_* ^2^ * = 0.071, β = 0.134);* ***Inspirate (P = <0.001, η_p_*** ^2^ *** = 0.734, β = 0.999)*** *; Drug × Inspirate (P = 0.165, η_p_* ^2^ * = 0.167, β = 0.274)*				
ΔcO_2_Hb (µmol·L^−1^)	0.0 ± 0.0	−5.5 ± 6.0	4.4 ± 8.3	−3.2 ± 6.6
*Drug (P = 0.102, η_p_* ^2^ * = 0.245, β = 0.370);* ***Inspirate (P = 0.005, η_p_*** ^2^ *** = 0.559, β = 0.893)*** *; Drug × Inspirate (P = 0.423, η_p_* ^2^ * = 0.065, β = 0.118)*				
ΔcHHb (µmol·L^−1^)	0.0 ± 0.0	3.3 ± 5.5	0.5 ± 8.2	5.1 ± 7.7
*Drug (P = 0.594, η_p_* ^2^ * = 0.029, β = 0.079);* ***Inspirate (P = 0.025, η_p_*** ^2^ *** = 0.409, β = 0.660)*** *; Drug × Inspirate (P = 0.523, η_p_* ^2^ * = 0.042, β = 0.092)*				
ΔctHb (µmol·L^−1^)	0.0 ± 0.0	−2.2 ± 10.6	4.9 ± 15.2	1.9 ± 13.7
*Drug (P = 0.264, η_p_* ^2^ * = 0.123, β = 0.188); Inspirate (P = 0.438, η_p_* ^2^ * = 0.061, β = 0.113); Drug × Inspirate (P = 0.854, η_p_* ^2^ * = 0.004, β = 0.053)*				
OI (%)	0.0 ± 0.0	−9.2 ± 5.9	2.8 ± 6.2	−9.5 ± 4.1
*Drug (P = 0.369, η_p_* ^2^ * = 0.074, β = 0.138);* ***Inspirate (P = <0.001, η_p_*** ^2^ *** = 0.879, β = 1.000)*** *; Drug × Inspirate (P = 0.182, η_p_* ^2^ * = 0.156, β = 0.256)*				

Values are mean ± SD; MCA_V_: middle cerebral artery blood velocity; sMCA_V_: systolic MCA_V_; dMCA_V_: diastolic MCA_V_; PI: pulsatility index; CVRi: cerebrovascular resistance index; CVCi: cerebrovascular conductance index; CDO_2_: cerebral delivery of oxygen; ΔcO_2_Hb: change in cortical oxy-hemoglobin concentration; ΔcHHb: change in cortical deoxy-hemoglobin concentration; ΔctHb: change in cortical total hemoglobin concentration; OI: oxygenation index. *P < 0.05 vs. normoxia – placebo; †P < 0.05 vs. normoxia – sildenafil. n = 12 for all parameters.

## Discussion

Taking a functionally integrative translational approach, the present study has identified two important findings. First, sildenafil selectively reduced systemic free-radical formation in hypoxia yet, contrary to original expectations, this was not accompanied by a reciprocal elevation in vascular NO bioavailability consistent with (attenuated) systemic OXNOS. Second, despite a selective reduction in MCAv that likely reflected enhanced (hypoxic) vasodilation given the attenuated hypoxia-induced cerebral deoxygenation and clearly independent of hyperventilation-induced hypocapnia-mediated cerebral vasoconstriction (i.e. PET_CO2_ invariant), sildenafil increased LF phase and restored the hypoxia-induced attenuation in CVR_CO2Hyper_. Collectively, these findings provide evidence for a PDE-5 inhibitory pathway that enhances select aspects of cerebrovascular function in hypoxia, subsequent to a systemic improvement in redox homeostasis that is independent of systemic NO bioavailability.

### Molecular function

Owing to the low reduction potential for the ${\text{A}}^{\cdot -}$ /ascorbate monanion (A ${\text{H}}^{-}$ ) couple, any oxidizing radical ( ${\text{R}}^{\cdot }$ ) present within the systemic/cerebral circulation will react with A ${\text{H}}^{-}$ to form ${\text{A}}^{\cdot -}$ ( ${\text{R}}^{\cdot }$ + A ${\text{H}}^{-}$ → $\;{\text{A}}^{\cdot -}$ + R-H, *E^0^* = 282 mV).^30^ The EPR spectroscopic detection of A^·−^ thus provides the first direct and unequivocal evidence for reduced systemic free radical formation in hypoxia following PDE-5 inhibition. Despite the inverse relationship observed between free-radical formation and NO bioavailability under conditions of acute, albeit more severe, hypoxia,^22,25,45^ this did not translate to a corresponding elevation in systemic NO bioavailability following sildenafil administration in hypoxia. Despite lower total plasma NO bioavailability observed under conditions of hypoxia (primarily attributed to lower ${\text{NO}}_{2}^{-}$ ), it should be noted that the vasodilatory properties of ${\text{NO}}_{2}^{-}$ in hypoxia are greatly enhanced.^46,47^ Coupled with sildenafil, a highly selective inhibitor of cGMP-specific PDE-5 that enhances NO-mediated smooth muscle relaxation, it is plausible that this may result in a ‘supercharged’ milieu to optimize cerebral bioenergetic homeostasis.

### Cerebrovascular function

The functional recovery of hypoxia-induced cerebrovascular impairments following sildenafil administration, taking the form of elevated CVR_CO2HYPER_ and a less pressure-passive dCA response (increased LF phase) are equally novel findings. Not only does sildenafil have the capacity to cross the blood-brain-barrier^6^ and act directly upon the central nervous system where PDE-5 has been localized to neurons and glial cells,^6^ sildenafil can also target PDE-5 within cerebrovascular endothelial cells.^9^ Accordingly, the NO/cGMP signal pathway has been shown to inhibit neuroinflammation, increase CBF and promote neurogenesis and synaptic plasticity.^48,49^

Additionally, these findings may be attributed to sympathetic hyperactivity following sildenafil administration in hypoxia,^50,51^ given that ganglion blockade with trimethaphan diminishes dCA^52^ and cerebral vasomotor reactivity^53^. Although sympathetic nervous activity (SNA) was not assessed directly in the present study, we did observe an elevation in heart rate with sildenafil, indicative of increased sympathetic tone.^54^ Equally, whilst we did not observe any changes in TFA Gain estimates in either the low or very-low frequency bands, the use of Phase estimates alone has been shown to be a more reliable measure of dCA than Gain in clinical studies.^43,55–57^

It should be noted that while our findings under conditions of normoxia are supported by previous research,^58^ there does exists a discrepancy with that of Jahshan, et al.^59^ who demonstrated sildenafil to increase CVR_CO2HYPER_ without affecting basal cerebrovascular tone or static/dynamic indices of CA. These discrepancies may be related to differences in drug dosage/timing notwithstanding technical nuances associated with the analytical assessment of CVR_CO2HYPER_ and dCA. In our study, we administered 50 mg of sildenafil in normoxia and employed a 3-min inhalation of 5% C ${\text{O}}_{2}$ with 21% ${\text{O}}_{2}$ (balanced nitrogen) for the assessment of CVR_CO2HYPER_ and 5-min of spontaneous oscillations in MAP/MCAv for dCA. In contrast, Jahshan, et al.^59^ combined 100 mg of sildenafil (in normoxia), with a carbogen mixture consisting of 5% C ${\text{O}}_{2}$ with 95% ${\text{O}}_{2}$ to assess CVR_CO2Hyper_ and graded intravenous infusions of phenylephrine to assess dCA. Extending prior research findings employing nitric oxide (NO) donation/blockade,^41,60,61^ Jahshan, et al.^59^ concluded that sildenafil-induced amplification of the NO-cGMP signaling pathway contributes solely to CVR_CO2_, without affecting the static and dynamic pressure-dependent mechanisms of CA. That we demonstrate tentative evidence of improved dCA [elevated (LF) Phase] is novel, albeit constrained by the interpretive deficiencies associated with spontaneous (as opposed to forced) oscillations and consistent with our prior contention that dCA is subject to redox-regulation.^24,45^ Further research is warranted to further explore functional integration of the NO-CVR_CO2_-CA axis.

### Clinical importance

The findings from this study are clinically relevant given that free radical-mediated impairments in cerebrovascular function caused by arterial hypoxaemia precede myriad infectious and degenerative neurological disorders.^62^ While this is the first investigation to explore the cerebrovascular implications of sildenafil under controlled conditions of acute inspiratory hypoxia utilizing a placebo-controlled cross-over design, these findings are commensurate with research in those with existing cerebrovascular impairments. In patients with pulmonary hypertension that are characterized by lower CBF and cerebrovascular reactivity, sildenafil enhanced visually-evoked flow responses indicating improved neurovascular coupling.^15^ More recently, a single dose (50 mg) of sildenafil has been shown to increase global CBF and CMR ${\text{O}}_{2}$ in patients with Alzheimer's Disease^10^ and increase CVR ${\text{CO}}_{2}$ in traumatic brain injury (TBI) patients.^63^ The protective effects of sildenafil in these patients could be attributed to lower oxidative stress and enhanced potentiation of endothelial derived NO in sufficiently damaged microvasculature proving to be the unifying mechanism to explain these clinically relevant findings.

### Limitations

The present study has several limitations that warrant consideration. First, MCAv represents an indirect surrogate measure of CBF and given that MCA diameter appears to increase in hypoxia^64^, we cannot rule out the possibility that we underestimated the volumetric changes incurred by hypoxia and sildenafil. In support, the selective reduction observed in MCAv during the sildenafil hypoxic trial may reflect an increase in CBF subsequent to enhanced vasodilation, highlighting the importance of duplex imaging of global CBF (flow and velocity metrics of internal carotid and vertebral arteries) in future studies. Second, there has been an on-going debate regarding the best method to quantity dCA and in particular, the use of spontaneous versus forced oscillations on blood pressure as the TFA input. The present study utilized spontaneous oscillations in blood pressure which is arguably more reflective of basal activity, safer to reproduce in a clinical population and importantly, does not challenge either the autonomic or cardiorespiratory systems.^65^ However, this approach assesses the minimal level of blood pressure variations leading to lower TFA coherence and diminished linearity within the cerebral pressure-flow relationship.^66^ Alternatively, it has been recommended that the TFA input should utilize forced oscillations in blood pressure (i.e. repeated squat-stands) to increase the signal-to-noise ratio,^43^ arguing that this reflects everyday challenges associated with large swings of blood pressure (i.e. bending down, standing up, walking upstairs) and that owing to the high coherence (e.g. close to 1.0), the changes in phase and gain are interpretable.^66^ However, we have consistently identified that >50% of young, healthy participants experience vasovagal syncope and associated cephalalgia during forced oscillations at an F_I_O_2_ of 0.12 (DM Bailey et al, unpublished observations), hence why we constrained our analyses to spontaneous oscillations only. Lastly, these findings are constrained to that of normobaric hypoxia with comparison to chronic high-altitude (hypobaric hypoxic) accordingly difficult owing to different physiological responses.^67,68^ However, we, among others, have demonstrated that acclimatization to high-altitude hypoxia increases basal systemic nitric oxide bioavailability^33,69^ which is responsible, at least in in part for, increasing cerebral substrate delivery and preserving bioenergetic function consistent with the conservation of mass principle.^70^ An improvement in cerebral redox homeostasis may represent the unifying mechanism that explains the functional improvements in cerebral oxygenation documented by Chan, et al.^18^ following sildenafil administration after chronic exposure to high-altitude.

## Conclusion

The present study has demonstrated that PDE-5 inhibition has the potential to restore redox homeostasis, namely attenuate systemic free radical formation without altering vascular NO bioavailability and recover select impairments in cerebral hemodynamic function following acute exposure to poikilocapnic hypoxia. These findings are clinically relevant given that free radical-mediated impairments in cerebrovascular function caused by arterial hypoxaemia precede myriad infectious and degenerative neurological disorders.

## Data Availability

Original data arising from this research are available directly from Professor Damian Miles Bailey upon reasonable request.

## References

[1] Ghofrani HA Osterloh IH Grimminger F. Sildenafil: from angina to erectile dysfunction to pulmonary hypertension and beyond. Nat Rev Drug Discov 2006; 5: 689–702. 16883306 10.1038/nrd2030 PMC7097805

[2] Jeremy JY Ballard SA Naylor AM , et al. Effects of sildenafil, a type-5 cGMP phosphodiesterase inhibitor, and papaverine on cyclic GMP and cyclic AMP levels in the rabbit corpus cavernosum in vitro. Br J Urol 1997; 79: 958–963. 9202566 10.1046/j.1464-410x.1997.00206.x

[3] Puzzo D Staniszewski A Deng SX , et al. Phosphodiesterase 5 inhibition improves synaptic function, memory, and amyloid-beta load in an Alzheimer's disease mouse model. J Neurosci 2009; 29: 8075–8086. 19553447 10.1523/JNEUROSCI.0864-09.2009 PMC6666028

[bibr4-0271678X251313747] Cuadrado-Tejedor M Hervias I Ricobaraza A , et al. Sildenafil restores cognitive function without affecting beta-amyloid burden in a mouse model of Alzheimer's disease. Br J Pharmacol 2011; 164: 2029–2041. 21627640 10.1111/j.1476-5381.2011.01517.x PMC3246665

[5] Orejana L Barros-Minones L Jordan J , et al. Sildenafil ameliorates cognitive deficits and tau pathology in a senescence-accelerated mouse model. Neurobiol Aging 2012; 33: e611-620–625.e20. 10.1016/j.neurobiolaging.2011.03.018 21546125

[6] Gómez‐Vallejo V Ugarte A García‐Barroso C , et al. Pharmacokinetic investigation of sildenafil using positron emission tomography and determination of its effect on cerebrospinal fluid cGMP levels. J Neurochem 2016; 136: 403–415. 26641206 10.1111/jnc.13454

[7] Sanderson TM Sher E. The role of phosphodiesterases in hippocampal synaptic plasticity. Neuropharmacology 2013; 74: 86–95. 23357335 10.1016/j.neuropharm.2013.01.011

[bibr8-0271678X251313747] Van Staveren WCG Steinbusch HWM Markerink-Van Ittersum M , et al. mRNA expression patterns of the cGMP-hydrolyzing phosphodiesterases types 2, 5, and 9 during development of the rat brain. J Comp Neurol 2003; 467: 566–580. 14624489 10.1002/cne.10955

[9] Teich AF Sakurai M Patel M , et al. PDE5 exists in human neurons and is a viable therapeutic target for neurologic disease. J Alzheimers Dis 2016; 52: 295–302. 26967220 10.3233/JAD-151104 PMC4927884

[10] Sheng M Lu H Liu P , et al. Sildenafil improves vascular and metabolic function in patients with Alzheimer's disease. J Alzheimers Dis 2017; 60: 1351–1364. 29036811 10.3233/JAD-161006 PMC5805465

[bibr11-0271678X251313747] Ding G Jiang Q Li L , et al. Angiogenesis detected after embolic stroke in rat brain using magnetic resonance T2*WI. Stroke 2008; 39: 1563–1568. 18356548 10.1161/STROKEAHA.107.502146 PMC2581408

[bibr12-0271678X251313747] Zhang RL Chopp M Roberts C , et al. Sildenafil enhances neurogenesis and oligodendrogenesis in ischemic brain of middle-aged mouse. PLoS One 2012; 7: e48141. 23118941 10.1371/journal.pone.0048141 PMC3485244

[13] Yazdani A Khoja Z Johnstone A , et al. Sildenafil improves brain injury recovery following term neonatal hypoxia-ischemia in male rat pups. Dev Neurosci 2016; 38: 251–263. 27614933 10.1159/000448327

[14] Kruuse C Gupta S Nilsson E , et al. Differential vasoactive effects of sildenafil and tadalafil on cerebral arteries. Eur J Pharmacol 2012; 674: 345–351. 22094063 10.1016/j.ejphar.2011.10.037

[15] Rosengarten B Schermuly RT Voswinckel R , et al. Sildenafil improves dynamic vascular function in the brain: studies in patients with pulmonary hypertension. Cerebrovasc Dis 2006; 21: 194–200. 16388195 10.1159/000090555

[16] Adesuyan M Jani YH Alsugeir D , et al. Phosphodiesterase type 5 inhibitors in men with erectile dysfunction and the risk of Alzheimer disease: a cohort study. Neurology 2024; 102: e209131. 38324745 10.1212/WNL.0000000000209131 PMC10890837

[17] Sikandaner HE Park SY Kim MJ , et al. Neuroprotective effects of sildenafil against oxidative stress and memory dysfunction in mice exposed to noise stress. Behav Brain Res 2017; 319: 37–47. 27836585 10.1016/j.bbr.2016.10.046

[18] Chan CWM Hoar H Pattinson K , et al. Effect of sildenafil and acclimatization on cerebral oxygenation at altitude. Clin Sci (Lond) 2005; 109: 319–324. 15865603 10.1042/CS20050036

[19] Koupparis AJ Jeremy JY Muzaffar S , et al. Sildenafil inhibits the formation of superoxide and the expression of gp47 NAD[P]H oxidase induced by the thromboxane A2 mimetic, U46619, in corpus cavernosal smooth muscle cells. BJU Int 2005; 96: 423–427. 16042742 10.1111/j.1464-410X.2005.05643.x

[20] Lundberg JO Weitzberg E Gladwin MT. The nitrate-nitrite-nitric oxide pathway in physiology and therapeutics. Nat Rev Drug Discov 2008; 7: 156–167. 18167491 10.1038/nrd2466

[21] Hollas MA Ben Aissa M Lee SH , et al. Pharmacological manipulation of cGMP and NO/cGMP in CNS drug discovery. Nitric Oxide 2019; 82: 59–74. 30394348 10.1016/j.niox.2018.10.006 PMC7645969

[22] Bailey DM Taudorf S Berg RMG , et al. Increased cerebral output of free radicals during hypoxia: implications for acute Mountain sickness? Am J Physiol Regul Integr Comp Physiol 2009; 297: R1283–1292. 19726713 10.1152/ajpregu.00366.2009

[23] Ogoh S Nakahara H Ueda S , et al. Effects of acute hypoxia on cerebrovascular responses to carbon dioxide. Exp Physiol 2014; 99: 849–858. 24632495 10.1113/expphysiol.2013.076802

[24] Bailey DM Brugniaux JV Filipponi T , et al. Exaggerated systemic oxidative-inflammatory-nitrosative stress in chronic mountain sickness is associated with cognitive decline and depression. J Physiol 2019; 597: 611–629. 30397919 10.1113/JP276898 PMC6332753

[25] Bailey DM Dehnert C Luks AM , et al. High-altitude pulmonary hypertension is associated with a free radical-mediated reduction in pulmonary nitric oxide bioavailability. J Physiol 2010; 588: 4837–4847. 20876202 10.1113/jphysiol.2010.194704 PMC3010150

[26] Bartsch P Swenson ER. Acute high-altitude illnesses. N Engl J Med 2013; 369: 1666–1667. 10.1056/NEJMc1309747 24152275

[27] WMA, World Medical Association. World medical association declaration of Helsinki: ethical principles for medical research involving human subjects. J Am Med Assoc 2013; 310: 2191–2194. 10.1001/jama.2013.281053 24141714

[28] Wang J Brown MA Tam SH , et al. Effects of diet on measurement of nitric oxide metabolites. Clin Exp Pharmacol Physiol 1997; 24: 418–420. 9171946 10.1111/j.1440-1681.1997.tb01212.x

[29] Nichols DJ Muirhead GJ Harness JA. Pharmacokinetics of sildenafil after single oral doses in healthy male subjects: absolute bioavailability, food effects and dose proportionality. Br J Clin Pharmacol 2002; 53 Suppl 1: 5S–12S. 11879254 10.1046/j.0306-5251.2001.00027.x PMC1874258

[30] Buettner GR Jurkiewicz BA. Ascorbate free radical as a marker of oxidative stress: an EPR study. Free Radic Biol Med 1993; 14: 49–55. 8384150 10.1016/0891-5849(93)90508-r

[31] Bailey DM Rasmussen P Evans KA , et al. Hypoxia compounds exercise-induced free radical formation in humans; partitioning contributions from the cerebral and femoral circulation. Free Radic Biol Med 2018; 124: 104–113. 29859345 10.1016/j.freeradbiomed.2018.05.090

[32] Bailey DM Rasmussen P Overgaard M , et al. Nitrite and S-nitrosohemoglobin exchange across the human cerebral and femoral circulation: Relationship to basal and exercise blood flow responses to hypoxia. Circulation 2017; 135: 166–176. 27881556 10.1161/CIRCULATIONAHA.116.024226

[33] Stacey BS Hoiland RL Caldwell HG , et al. Lifelong exposure to high-altitude hypoxia in humans is associated with improved redox homeostasis and structural-functional adaptations of the neurovascular unit. J Physiol 2023; 601: 1095–1120. 36633375 10.1113/JP283362 PMC10952731

[34] Rogers SC Khalatbari A Gapper PW , et al. Detection of human red blood cell-bound nitric oxide. J Biol Chem 2005; 280: 26720–26728. 15879596 10.1074/jbc.M501179200

[35] Jellema WT Wesseling KH Groeneveld AB , et al. Continuous cardiac output in septic shock by simulating a model of the aortic input impedance: a comparison with bolus injection thermodilution. Anesthesiology 1999; 90: 1317–1328. 10319780 10.1097/00000542-199905000-00016

[36] Willie CK Colino FL Bailey DM , et al. Utility of transcranial doppler ultrasound for the integrative assessment of cerebrovascular function. J Neurosci Methods 2011; 196: 221–237. 21276818 10.1016/j.jneumeth.2011.01.011

[37] Woodside JDS Gutowski M Fall L , et al. Systemic oxidative-nitrosative-inflammatory stress during acute exercise in hypoxia; implications for microvascular oxygenation and aerobic capacity. Exp Physiol 2014; 99: 1648–1662. 25344270 10.1113/expphysiol.2014.081265

[38] Jasper HH. The ten‐twenty electrode system of the international federation. Electroencephalogr Clin Neurophysiol 1958; 10: 371–375. 10590970

[39] Duncan A Meek JH Clemence M , et al. Optical pathlength measurements on adult head, calf and forearm and the head of the newborn infant using phase resolved optical spectroscopy. Phys Med Biol 1995; 40: 295–304. 7708855 10.1088/0031-9155/40/2/007

[40] van der Zee P Cope M Arridge SR , et al. Experimentally measured optical pathlengths for the adult head, calf and forearm and the head of the newborn infant as a function of inter optode spacing. Adv Exp Med Biol 1992; 316: 143–153. 1288074 10.1007/978-1-4615-3404-4_17

[41] Zhang R Zuckerman JH Giller CA , et al. Transfer function analysis of dynamic cerebral autoregulation in humans. Am J Physiol 1998; 274: H233–241. 9458872 10.1152/ajpheart.1998.274.1.h233

[42] Claassen JA Meel-van den Abeelen AS Simpson DM , et al. Transfer function analysis of dynamic cerebral autoregulation: a white paper from the international cerebral autoregulation research network. J Cereb Blood Flow Metab 2016; 36: 665–680. 26782760 10.1177/0271678X15626425 PMC4821028

[43] Panerai RB Brassard P Burma JS , et al. Transfer function analysis of dynamic cerebral autoregulation: a CARNet white paper 2022 update. J Cereb Blood Flow Metab 2023; 43: 3–25. 35962478 10.1177/0271678X221119760 PMC9875346

[44] Bailey DM Rimoldi SF Rexhaj E , et al. Oxidative-nitrosative stress and systemic vascular function in highlanders with and without exaggerated hypoxemia. Chest 2013; 143: 444–451. 22922469 10.1378/chest.12-0728

[45] Bailey DM Evans KA James PE , et al. Altered free radical metabolism in acute mountain sickness: implications for dynamic cerebral autoregulation and blood-brain barrier function. J Physiol 2009; 587: 73–85. 18936082 10.1113/jphysiol.2008.159855 PMC2670024

[46] Maher AR Milsom AB Gunaruwan P , et al. Hypoxic modulation of exogenous nitrite-induced vasodilation in humans. Circulation 2008; 117: 670–677. 18212289 10.1161/CIRCULATIONAHA.107.719591

[47] Cosby K Partovi KS Crawford JH , et al. Nitrite reduction to nitric oxide by deoxyhemoglobin vasodilates the human circulation. Nat Med 2003; 9: 1498–1505. 14595407 10.1038/nm954

[48] Garthwaite G Hampden-Smith K Wilson GW , et al. Nitric oxide targets oligodendrocytes and promotes their morphological differentiation. Glia 2015; 63: 383–399. 25327839 10.1002/glia.22759 PMC4309495

[49] Raposo C Nunes A. K d S Luna R. L d A , et al. Sildenafil (viagra) protective effects on neuroinflammation: the role of iNOS/NO system in an inflammatory demyelination model. Mediators Inflamm 2013; 2013: 321460. 23970812 10.1155/2013/321460 PMC3736464

[50] Phillips BG Kato M Pesek CA , et al. Sympathetic activation by sildenafil. Circulation 2000; 102: 3068–3073. 11120696 10.1161/01.cir.102.25.3068

[51] Steinback CD Salzer D Medeiros PJ , et al. Hypercapnic vs. hypoxic control of cardiovascular, cardiovagal, and sympathetic function. Am J Physiol Regul Integr Comp Physiol 2009; 296: R402–410. 19091913 10.1152/ajpregu.90772.2008

[52] Zhang R Zuckerman JH Iwasaki K , et al. Autonomic neural control of dynamic cerebral autoregulation in humans. Circulation 2002; 106: 1814–1820. 12356635 10.1161/01.cir.0000031798.07790.fe

[53] Przybyłowski T Bangash MF Reichmuth K , et al. Mechanisms of the cerebrovascular response to apnoea in humans. J Physiol 2003; 548: 232. 10.1113/jphysiol.2002.029678 PMC2342799 12588894

[54] Grassi G Vailati S Bertinieri G , et al. Heart rate as marker of sympathetic activity. J Hypertens 1998; 16: 1635–1639. 9856364 10.1097/00004872-199816110-00010

[55] Intharakham K Beishon L Panerai RB , et al. Assessment of cerebral autoregulation in stroke: a systematic review and meta-analysis of studies at rest. J Cereb Blood Flow Metab 2019; 39: 2105–2116. 31433714 10.1177/0271678X19871013 PMC6827119

[bibr56-0271678X251313747] Sheriff F Castro P Kozberg M , et al. Dynamic cerebral autoregulation post endovascular thrombectomy in acute ischemic stroke. Brain Sci 2020; 10: 641. 32948073 10.3390/brainsci10090641 PMC7564150

[57] van Beek AH Claassen JA Rikkert MG , et al. Cerebral autoregulation: an overview of current concepts and methodology with special focus on the elderly. J Cereb Blood Flow Metab 2008; 28: 1071–1085. 18349877 10.1038/jcbfm.2008.13

[58] Kruuse C Hansen AE Larsson HB , et al. Cerebral haemodynamic response or excitability is not affected by sildenafil. J Cereb Blood Flow Metab 2009; 29: 830–839. 19209179 10.1038/jcbfm.2009.10

[59] Jahshan S Dayan L Jacob G. Nitric oxide-sensitive guanylyl cyclase signaling affects CO(2)-dependent but not pressure-dependent regulation of cerebral blood flow. Am J Physiol Regul Integr Comp Physiol 2017; 312: R948–R955. 28356297 10.1152/ajpregu.00241.2016

[60] Zhang R Wilson TE Witkowski S , et al. Inhibition of nitric oxide synthase does not alter dynamic cerebral autoregulation in humans. Am J Physiol Heart Circ Physiol 2004; 286: H863–869. 15008160 10.1152/ajpheart.00373.2003

[61] Lavi S Egbarya R Lavi R , et al. Role of nitric oxide in the regulation of cerebral blood flow in humans: chemoregulation versus mechanoregulation. Circulation 2003; 107: 1901–1905. 12665500 10.1161/01.CIR.0000057973.99140.5A

[62] Kisler K Nelson AR Montagne A , et al. Cerebral blood flow regulation and neurovascular dysfunction in Alzheimer disease. Nat Rev Neurosci 2017; 18: 419–434. 28515434 10.1038/nrn.2017.48 PMC5759779

[63] Kenney K Amyot F Moore C , et al. Phosphodiesterase-5 inhibition potentiates cerebrovascular reactivity in chronic traumatic brain injury. Ann Clin Transl Neurol 2018; 5: 418–428. 29687019 10.1002/acn3.541 PMC5899908

[64] Wilson MH Edsell MEG Davagnanam I , et al. Cerebral artery dilatation maintains cerebral oxygenation at extreme altitude and in acute hypoxia—an ultrasound and MRI study. J Cereb Blood Flow Metab 2011; 31: 2019–2029. 21654697 10.1038/jcbfm.2011.81 PMC3208157

[65] Tzeng YC Panerai RB. CrossTalk proposal: dynamic cerebral autoregulation should be quantified using spontaneous blood pressure fluctuations. J Physiol 2018; 596: 3–5. 29207213 10.1113/JP273899 PMC5746519

[66] Smirl JD Hoffman K Tzeng YC , et al. Methodological comparison of active- and passive-driven oscillations in blood pressure; implications for the assessment of cerebral pressure-flow relationships. J Appl Physiol (1985) 2015; 119: 487–501. 26183476 10.1152/japplphysiol.00264.2015 PMC4556839

[67] Ainslie PN Hoiland RL Bailey DM. Lessons from the laboratory; integrated regulation of cerebral blood flow during hypoxia. Exp Physiol 2016; 101: 1160–1166. 27058994 10.1113/EP085671

[68] Hoiland RL Bain AR Rieger MG , et al. Hypoxemia, oxygen content, and the regulation of cerebral blood flow. Am J Physiol Regul Integr Comp Physiol 2016; 310: R398–413. 26676248 10.1152/ajpregu.00270.2015 PMC4796739

[69] Beall CM Laskowski D Erzurum SC. Nitric oxide in adaptation to altitude. Free Radic Biol Med 2012; 52: 1123–1134. 22300645 10.1016/j.freeradbiomed.2011.12.028 PMC3295887

[70] Bailey DM Bain AR Hoiland RL , et al. Severe hypoxaemic hypercapnia compounds cerebral oxidative-nitrosative stress during extreme apnoea: implications for cerebral bioenergetic function. J Physiol 2024; 602: 5659–5684. 38348606 10.1113/JP285555

